# Development of LTCC-packaged optocouplers as optical galvanic isolation for high-temperature applications

**DOI:** 10.1038/s41598-022-15631-7

**Published:** 2022-07-08

**Authors:** Pengyu Lai, David Gonzalez, Syam Madhusoodhanan, Abbas Sabbar, Salahaldein Ahmed, Binzhong Dong, Jiangbo Wang, H. Alan Mantooth, Shui-Qing Yu, Zhong Chen

**Affiliations:** 1 Department of Electrical Engineering, University of Arkansas, Fayetteville, AR 72701 USA; 2 Key Laboratory of Wide Bandgap Semiconductor Materials and Devices, 233 Sufu Road, Yiwu, Zhejiang 322009 People’s Republic of China

**Keywords:** Electrical and electronic engineering, Optical techniques

## Abstract

This paper reports high-temperature optocouplers for signal galvanic isolation. Low temperature co-fired ceramic (LTCC) technology was used in the design and fabrication of the high-temperature optocoupler package. The optimal coupling behaviors, driving capabilities and response speed of the optocouplers were concentrated and investigated in this paper. Emitters and detectors with different emission and spectral wavelengths were studied to achieve optimal coupling behaviors. Relatively high coupling efficiency is achieved with emitters and detectors of emission and spectral wavelength in the red spectrum (i.e., 620–750 nm), leading to higher current transfer ratios (CTR). To further enhance the electrical performance, optocouplers with multiple detectors in parallel were designed and fabricated. CTR, leakage current and response speed (i.e., propagation delay, rise time and fall time) of the optocouplers were characterized over a range of temperatures from 25 to 250 °C. The CTR degrades at high temperatures, while the leakage current and response speed show little degradation with varying temperatures. Furthermore, the behaviors of the optocouplers with varying temperatures are modeled and analyzed.

## Introduction

The temperature tolerance of semiconductor devices and integrated circuits (ICs) is greatly improved due to the revolution of semiconductor materials (e.g., silicon carbide (SiC) and gallium nitride (GaN))^1^ and technique (e.g., silicon on insulator (SOI))^2^. This allows power devices, mixed-signal circuits and control systems to be operated in high-temperature environments^3–5^. Since these applications involve high voltages, common-mode signals and fluctuating ground potentials, galvanic isolation devices are required as a protection method^6^. For example, in power systems, galvanic isolation devices and circuits are required for the gate driver circuitry to isolate the low-voltage logic controllers from the high-voltage components^7,8^. Among galvanic isolators, optocouplers, capacitors and transformers are commonly used^7–10^. Optocouplers provide a small packaging size, few connection components, low input drive currents and low power dissipation, making them more desirable than regular isolation transformers^8^. However, the performance degradation of optocouplers at elevated temperatures limits their applications in high-temperature environments^10^. Table 1 summarizes recently published optocouplers. Although some of them show high CTR, very few are capable of operating over 150 °C. Therefore, the design and fabrication of high-temperature optocouplers for galvanic isolation are highly desired.

**Table 1:** Recently published optocouplers.

Reference	^11^	^12^	^13^	^14^	^15^
CTR	2	0.002	0.02	0.008	1.6
Temperature	25 °C	25 °C	25 °C	25 °C	125 °C

A few studies on optoelectronic materials and devices for high-temperature applications were conducted and reported in recent years^16–21^. The spontaneous emission quantum efficiency (QE) of different light-emitting diode (LED) materials (i.e., indium-gallium-nitride-based (InGaN-based) multiple quantum wells (MQWs)) over a wide range of temperature was studied using photoluminescence (PL) measurements^17^. The InGaN-based MQW structures exhibit minimum QE drop at temperatures higher than 200 °C. The study was extended to other LED materials as well to investigate the QE at high temperatures. Sabbar et al.^18^ reported InGaN-based and aluminum-gallium-indium-phosphide-based (AlGaInP-based) MQW structures with different peak wavelength (i.e., 450 nm, 470 nm and 630 nm) for high-temperature optoelectronic applications. Moreover, further optimization into InGaN-based structures was proposed to enhance their behaviors at high temperatures, and relatively high QE at high temperatures (i.e., > 200 °C) is observed^19^. The temperature and injected current-dependent internal quantum efficiency (IQE) of InGaN-based MQW LEDs with different peak wavelengths (i.e., 450 nm, 470 nm and 530 nm) were studied using electroluminescence (EL) measurements^15^. Stable peak IQE of these LEDs at high temperatures was reported. These studies^17–20^ prove that AlGaN-based and AlGaInP-based MQW structures can be utilized to form LED devices in high-temperature optoelectronic applications. In addition, the spectral responsivity (SR) of InGaN-based MQW structures was investigated with the temperature range of − 200 °C to 500 °C^21^. The results indicate that the photodiodes can be used in high-temperature optocouplers. Although systematic studies of the optoelectronic devices for high-temperature applications were carried out, the LEDs and photodiodes were investigated individually. The high-temperature optical coupling behavior of the LEDs and photodiodes is not yet investigated. Therefore, high-temperature optocouplers, which integrate LEDs as emitters and photodiodes as detectors, need further studies.

In this paper, we report high-temperature optocouplers for optical galvanic isolation, which are capable of operating at 250 °C. The design was focused on the investigation and optimization of driving capability and response speed. Commercial high-temperature LEDs, which were studied in previous work^17–21^, are used as emitters and detectors in the optocouplers to investigate their optical coupling behaviors. Low temperature co-fired ceramic (LTCC) was utilized for the packaging of the optical isolator. The multilayer fabrication process of the LTCC process allows for easiness to create cavities for light to travel. In addition, LTCC allows for temperature stability and guarantees functionality to temperatures higher than conventional package technologies. The current transfer ratio (CTR), leakage current, propagation delay, rise time and fall time of the LTCC-based high-temperature optocouplers are measured over a range of temperatures from 25 to 250 °C. Device modeling is also provided to illustrate the behaviors of the optocouplers with varying temperatures.

## Device packaging and fabrication

Optocouplers are preferred to isolation transformers because they can provide galvanic isolation with a significantly reduced form factor and weight. Optocouplers often use dual in-line package (DIP), surface-mount technology (SMT) and small-outline package (SOP) types of packages^22,23^. These packages are normally made with epoxy-based materials, which do not endure wide temperature variations^24^. LTCC, on the other hand, is capable of withstanding an electrical operating temperature higher than 400 °C after firing^25^, which makes LTCC a promising packaging material for high-temperature applications. Moreover, LTCC technology utilizes a multilayer fabrication process, which allows for creating vias interconnect, cavities, and embedded traces. This makes the LTCC-based devices easy to be integrated with electrical circuits and systems, such as gate driver circuits and power modules. The fabrication for thick film ceramics, such as LTCC, has been successfully mastered in the High-Density Electronic Center (HiDEC) at the University of Arkansas^26^. This has increased the motivation for evaluating LTCC as a substrate or housing material for electronic devices. Table 2 shows a material composition comparison for common ceramics that are used for electronic housing, including LTCC. When comparing the different ceramics, temperature functionality, feasibility for tooling, mechanical strength, coefficient of thermal expansion (CTE) and other aspects must be considered. Even though other ceramics such as aluminum oxide (Al_2_O_3_) and silicon nitride (Si_3_N_4_) tend to be cheaper, extra process to achieve metal plating significantly increases the ceramic prize. In addition, AlN, Al_2_O_3_ and Si_3_N_4_ required a laser process to create the channel for the light to travel, which increases the risk of ceramic cracking. From this point of view, LTCC has a much greater advantage since its cavity is formed during ceramic fabrication. Therefore, an LTCC-based package was designed to encapsulate the emitters and detectors.

**Table 2:** Ceramic material comparison.

Material	AlN	Al_2_O_3_	Si_3_N_4_	LTCC (DuPont™ 951 Green Tape™)
Thermal Conductivity (W/mK)	180	18	30	4.6
CTE (ppm)	4.5	18	3.3	5.8
Elastic Strength (MPa)	330	300	310	230
Dielectric Strength kV/mm	17	14.6	14	7.8
Tooling (Cavity formation)	Laser grooving			Embedded in fabrication

**Figure 1:**
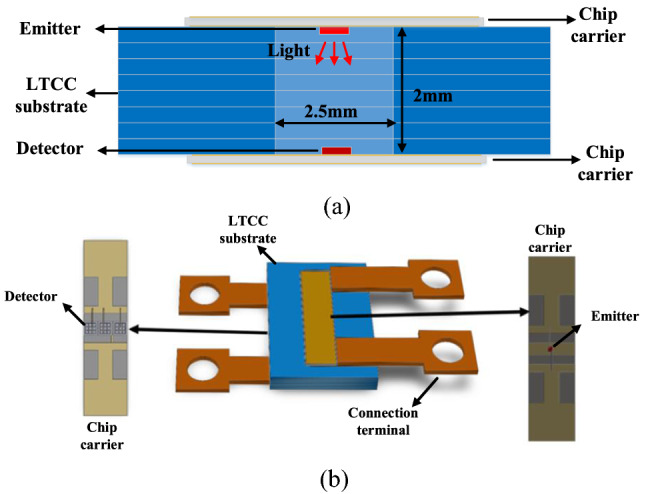
(**a**) Cross-sectional view and (**b**) 3D model view of the LTCC-based high-temperature optocoupler.

Figure 1a,b show the cross-sectional view and three-dimensional (3D) view of the LTCC-based high-temperature optocoupler. The package of the optocoupler consists of an LTCC-based substrate to support two chip carriers. The chip carriers, which are made by AlN-based gold-plated direct bonded copper (DBC), are utilized to hold the emitters asnd detectors and attached facing each other to the LTCC-based substrate (Fig. 1a). This allows for a free space transmission between the emitters and the detectors. After the emitters and detectors were encapsulated, copper leads were attached to the LTCC substrate as the connection terminals (Fig. 1b). It should be noted that the connection terminals are designed only for standalone optocoupler measurements and not to be integrated into application circuits.

**Figure 2:**
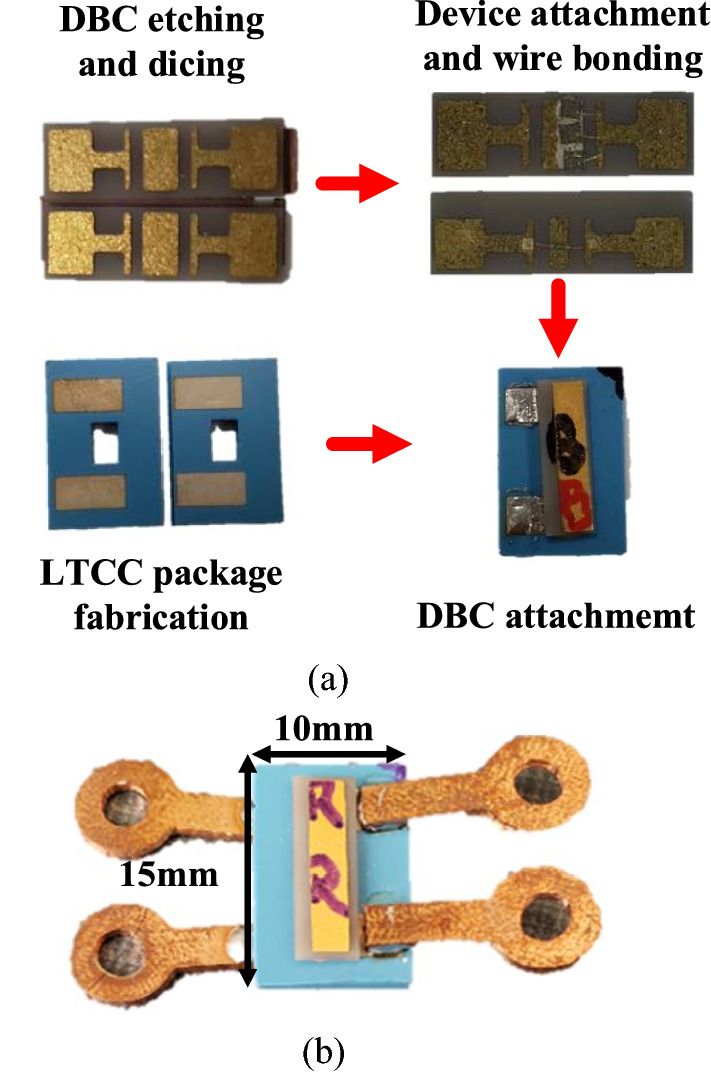
LTCC-based high-temperature optocoupler (**a**) fabrication flow and (**b**) fabricated sample.

Figure 2a shows the detailed fabrication flow of the LTCC-based high-temperature optocoupler. The LTCC packages and AlN-based gold-plated DBC were fabricated. The DBC was etched with a chemical solution to form a connection pattern and diced into pieces as chip carriers. The devices were attached to the middle section of the DBC pattern, and wire-bonding was performed to connect the device anode and cathode to DBC pads. After the wire bonding, the chip carriers are attached facing each other in the LTCC-based substrate by using high-temperature conductive epoxy (i.e., CW2400 by Chemtronics). The epoxy, which has a dropping point of 343 °C, provides a close sealing between chip carriers and packages, limiting the light from escaping. In addition, since the CTEs of AlN and LTCC substrate are closed (Table 2), the package has high mechanical stability and better performance at elevated temperatures. Finally, copper leads were attached by high-temperature solder alloy (i.e., SAC305). The fabricated LTCC-based high-temperature optocoupler is shown in Fig. 2b. The device has a length of 15 mm, width of 10 mm and thickness of 2 mm. InGaN-based, AlGaInP-based and aluminum-gallium-arsenide-based (AlGaAs-based) high-temperature commercial LEDs^27,28^ are utilized as the emitter and detector of the optocouplers since they are promising for the high-temperature optoelectronic device fabrications^17–21^. Table 3 shows the LED materials, peak wavelength, device dimensions and manufacturers of the LEDs that are packaged into the optocouplers. Optocouplers with different combinations of emitters and detectors were fabricated and characterized. The optocoupler samples were named based on emitter-detector combination, i.e., GD-BD stands for optocoupler sample with green for display as the emitter and blue for display as the detector. The fabricated optocouplers are shown in Table 4.

**Table 3:** Materials, peak wavelength, dimensions and manufacturers of the LEDs that are packaged.

Type of LEDs	LED Material	Peak wavelength	Bare die device dimension	Manufacturer
Blue for light (BL)	Indium gallium nitride (InGaN)	450 nm	1345 µm × 800 µm	HC Semitek
Blue for display (BD)	Indium gallium nitride (InGaN)	470 nm	191 µm × 270 µm	HC Semitek
Green for display (GD)	Indium gallium nitride (InGaN)	530 nm	191 µm × 270 µm	HC Semitek
Red for display (RD)	Aluminum gallium indium phosphide (AlGaInP)	630 nm	300 µm × 300 µm	HC Semitek
OPC-6900-21 (OPC)	Aluminum gallium arsenide (AlGaAs)	700 nm	960 µm × 960 µm	Marktech

**Table 4:** Fabricated LTCC-based high-temperature optocouplers.

Optocoupler	Emitter	Detector
BD-BD	Blue for display	Blue for display
BD-GD	Blue for display	Green for display
GD-GD	Green for display	Green for display
GD-BD	Green for display	Blue for display
GD-BL	Green for display	Blue for light
BL-GD	Blue for light	Green for display
RD-RD	Red for display	Red for display
RD-OPC	Red for display	OPC-6900-21

## Experiments and results

**Figure 3:**
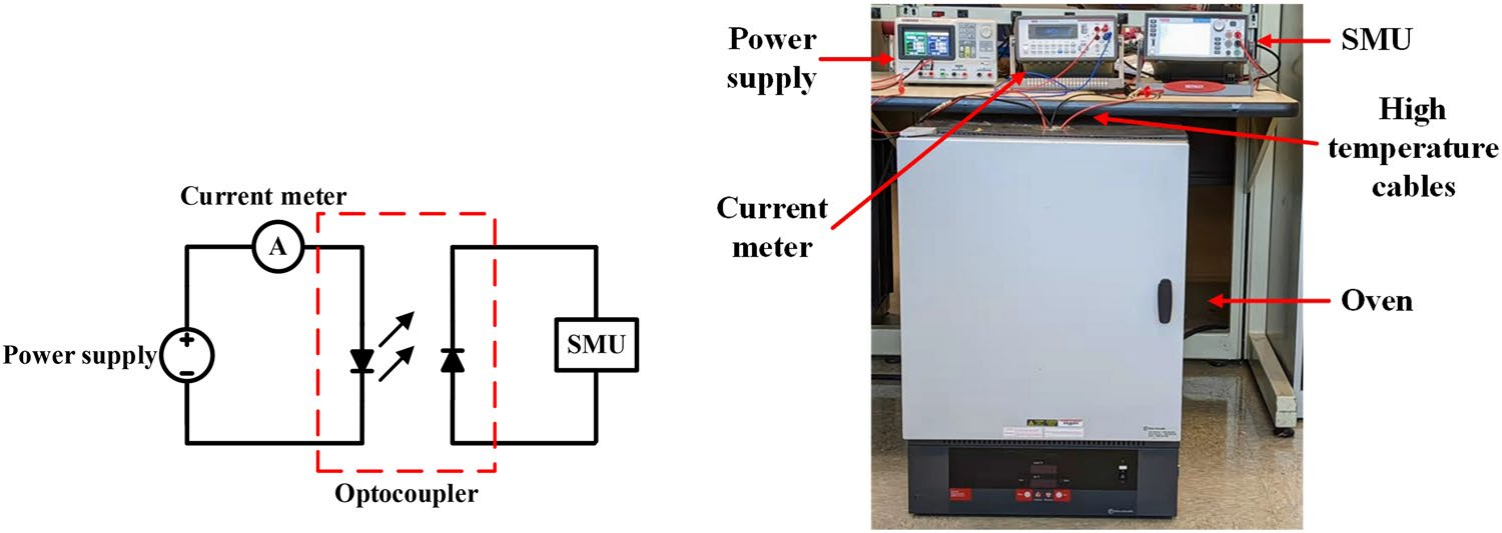
(**a**) Schematic and (**b**) experimental setup for DC characteristics.

As a galvanic isolation device, an optocoupler needs to provide sufficient output current to drive the next-stage circuit, such as a transimpedance amplifier (TIA). To understand the output performance of fabricated LTCC-based optocouplers, the output current and the CTR of the optocouplers were characterized with varying temperatures from 25 to 250 °C. Figure 3a,b show the schematic and experimental setup of the DC characterization for the LTCC-based high-temperature optocouplers. Siglent SPD3303X-E power supply is used to drive the emitter, and a current meter is used to measure the input current. Keithley 2450 source measure unit (SMU) is connected with the detector of the high-temperature optocoupler to measure its output current. The input current is controlled by changing the forward-bias voltage of the emitter and limited under the maximum forward current of the emitters (i.e., 30 mA). The optocouplers were characterized in Fisher Scientific 650–126 high-temperature oven to observe their performance with different temperatures. High-temperature cables are used for the connection between the optocouplers and the measuring instruments.

**Figure 4:**
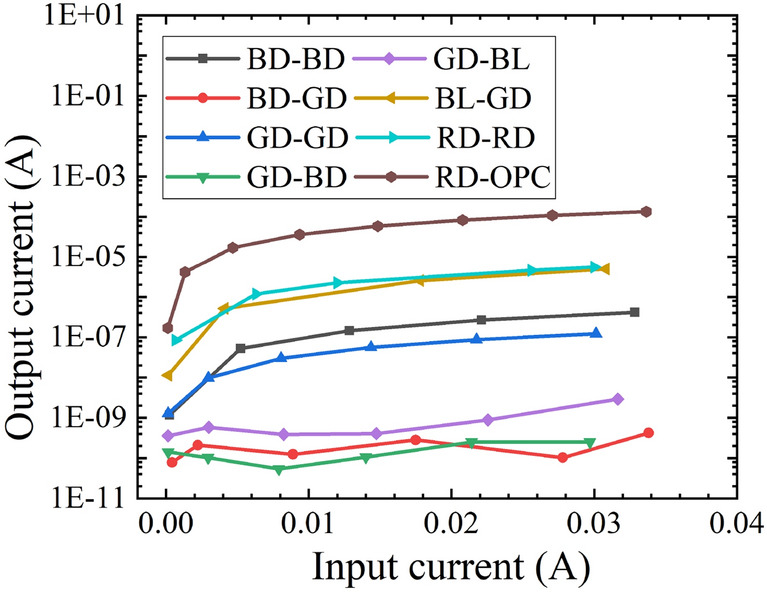
Output current versus input current of optocuplers with various types of emitters and detectors.

In order to determine the optical coupling behaviors and the coupling efficiency of emitters and detectors, optocouplers with various types of emitters and detectors were characterized. Figure 4 shows the output current versus the input current of the optocouplers at 25 °C. The highest output current is observed on RD-OPC. The output current is 134 µA when the input current is 30 mA. This is mainly because the OPC has the largest device size (Table 2). RD-RD and BL-GD also show good matches. The output currents of RD-RD and BL-GD are 5.5 µA and 5 µA when the input current is 30 mA. Other optocouplers (i.e., BD-BD, BD-GD, GD-GD, GD-BD and GD-BL) show relatively low output current (i.e., lower than 0.5 µA). This indicates that they are not suitable combinations to form optocouplers.

**Figure 5:**
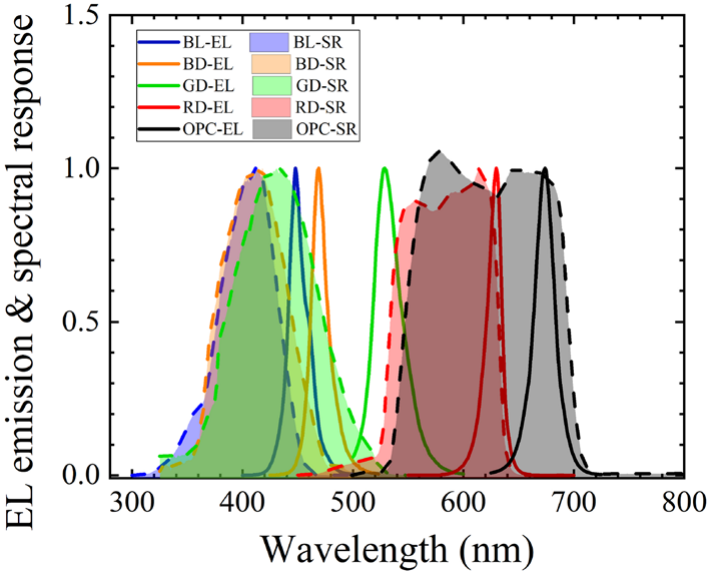
EL emissions and spectral responses for various types of emitters and detectors.

To further investigate the optical coupling behaviors of the emitters and detectors, the EL emission and SR measurements of the devices were carried out. The normalized EL emission and SR results are plotted together in Fig. 5. The EL emission of RD is from 600 to 650 nm, and the SR of OPC is from 550 to 700 nm. Therefore, the optocoupler with RD as the emitter and OPC as the detector shows a good match and relatively high output current (Fig. 4). As shown in Fig. 5, RD-RD and BL-GD also have overlap regions on the EL emission and SR, which makes them suitable combinations for optocouplers.

**Figure 6:**
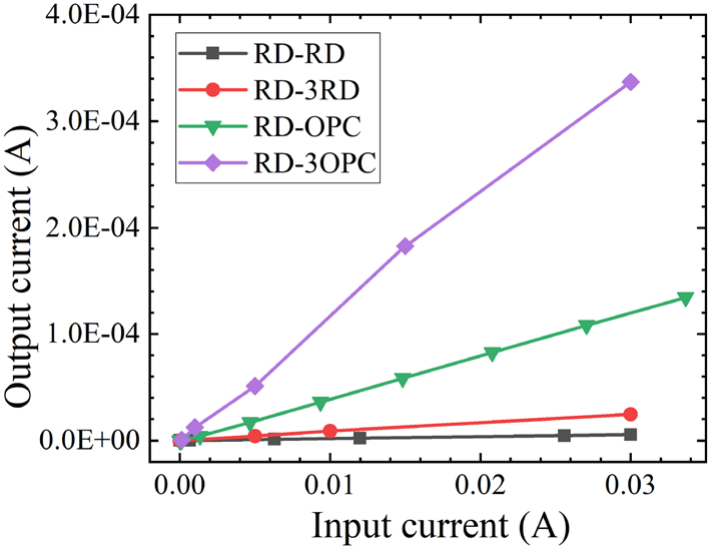
Optocoupler output current versus input current at 25 °C with different quantity of detectors.

RD-OPC and RD-RD samples exhibited better optical coupling behaviors than other optocouplers (e.g., BD-BD, BD-GD and GD-GD). Although RD-OPC has an output current higher than 100 µA, a higher output current is desired to drive external amplification circuits and achieve a better signal-to-noise ratio (SNR). The output current is strongly related to the total device area of detectors. Therefore, optocouplers with multiple detectors connected in parallel were fabricated to further enhance the output current. Table 5 summarizes the details of the fabricated optocoupler samples. The output current versus the input current of optocouplers with different numbers of parallel detectors is shown in Fig. 6. Increasing the quantity of the detectors enables efficient accommodation of the emitter beam area leading to higher output current. RD-3OPC shows the output current of 337 µA when the input current is 30 mA, which is ~ 2.6 times higher than RD-OPC (i.e., 134 µA). RD-3RD shows the output current of ~ 24 µA when the input current is 30 mA.

**Table 5:** Emitter and detector of fabriacted optocoupler samples.

Optocoupler	Emitter	Quantity of emitters in parallel	Detector	Quantity of detectors in parallel
RD-RD	RD	1	RD	1
RD-3RD	RD	1	RD	3
RD-OPC	RD	1	OPC	1
RD-3OPC	RD	1	OPC	3

**Figure 7:**
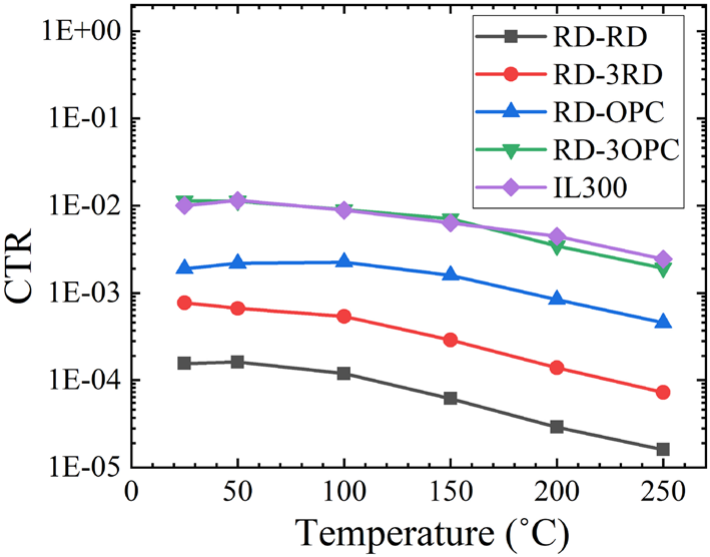
CTR of optocouplers with varying temperatures.

High-temperature characterization of the optocouplers was conducted in a high-temperature oven (Fig. 3b). As the ambient temperature increases, the output current and the CTR degrades due to the drop in the EL intensity of the emitters^29^. Figure 7 shows the measured CTR of the optocouplers with temperatures ranging from 25 to 250 °C. A commercial optocoupler (IL300 by Vishay)^22^ was also characterized to compare the thermal stability with the fabricated LTCC-based optocouplers. The RD-3OPC and the IL300 have similar CTR values at 25 °C (i.e., ~ 0.01). This indicates that the RD-3OPC has the potential to be operated as a galvanic isolation device. The CTRs of the optocouplers start to degrade when the temperature is over 100 °C.

**Figure 8:**
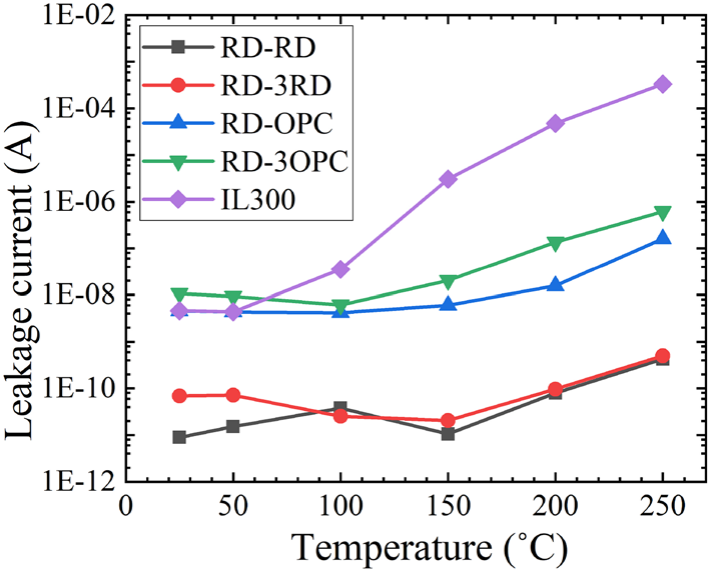
Leakage current of optocouplers with varying temperatures.

In real applications, optocouplers often operate with a small reverse bias voltage. The small reverse-bias voltage (e.g., − 1 V) increases the depletion width of the detector, which improves the number of photons absorption and increases the photocurrent. However, the rise in temperature also increases the reverse-bias current (i.e., leakage current) of the detectors, which is attributed to the thermal ionization^21^. The increase in the leakage current elevates the dark current noise power of the detector and limits the SNR of the circuit^30^. Furthermore, the variation of the leakage current with temperature shifts the quiescent point (Q-point) of the next stage circuit, which may cause mis-triggers. The leakage current of the optocouplers under − 1 V bias was measured with varying temperatures and shown in Fig. 8. It is observed that the commercial optocoupler IL300 shows a rapid increase in leakage current at temperatures above 100 °C. The leakage current of IL300 is ~ 100 µA at 250 °C, which is in the same magnitude as the photocurrent, indicating low SNR. The leakage current of RD-RD and RD-3RD does not show much variation with the increase of temperature. The leakage current of RD-RD and RD-3RD is ~ 0.5 nA at 250 °C. The leakage current of RD-3OPC increases from 10 nA at 25 °C to 600 nA at 250 °C. It should be noted that the photocurrent of RD-3OPC is ~ 50 µA at 250 °C, which is two orders of magnitude higher than the leakage current.

**Figure 9:**
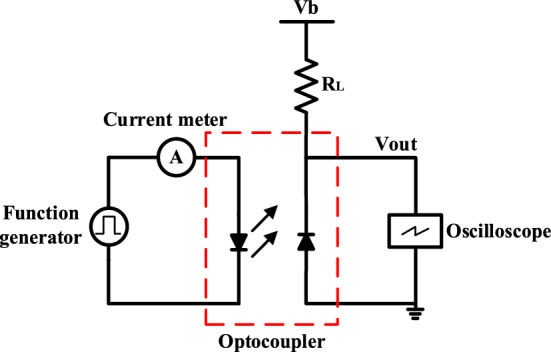
Optocoupler test circuit for transient characteristics.

The response time (i.e., propagation delay, rise time and fall time) of the optocoupler determines its bandwidth. Thus, transient measurements of the optocouplers were conducted to characterize their propagation delay, rise time and fall time. Figure 9 shows the test circuit for transient characteristics of the optocouplers. A Rigol DG1022 function generator is used to generate the pulse-width modulation (PWM) signal for the optocouplers. A Tektronix TDS2012B oscilloscope is utilized to capture the output signals of the optocouplers. A 15 kΩ resistor (R_L_) is connected to the cathode of the detector. To avoid the over-heating of the emitters at high temperatures, the input current was limited at 20 mA with an input PWM voltage of 2.5 V. The optocouplers were measured at a reverse-bias voltage (V_b_) of − 1 V to improve the signal detection.

**Figure 10:**
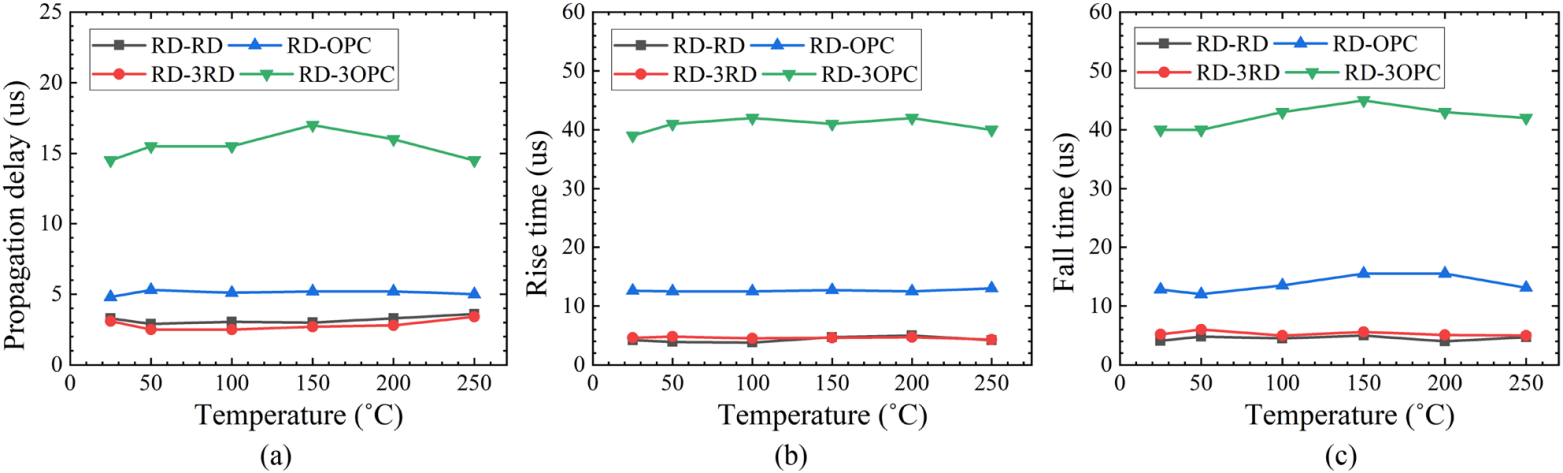
Transient measurement results of optocouplers with varying temperatures: (**a**) propagation delay, (**b**) rise time, and (**c**) fall time.

The transient measurement results of four optocouplers with varying temperatures are shown in Fig. 10. The propagation delay, rise time and fall time show low-temperature sensitivity when the temperature increases from 25 to 250 °C. The fall time of the optocouplers shows the same magnitude as the rise time (Fig. 10b,c). It is observed that the response time is steady when replacing one RD by three RDs (as the detector). Meanwhile, the response time of RD-3OPC is around three times higher than RD-OPC.

## Discussion

It is observed that the performance of the LTCC-based optocouplers varies with temperatures. The CTR shows degradation when the temperature is higher than 100 °C. This is because the light output power, *P*, of the emitters drops at high-temperature conditions. Based on the power-law relationship, the light output power of the emitters is proportional to the integrated EL signal (*L*_*EL*_)^29,31^. The relationship can be described as:

1 $${L}_{EL}({I}_{in}, T)\propto P({I}_{in}, T)$$

where *I*_*in*_ is the input current of emitters, and *T* is the temperature.

For detectors, the relationship between the light input power (i.e., the light output power of the emitters) and the photocurrent (*I*_*ph*_) is^32^:

2 $$R\left(\lambda , T\right)=\frac{{I}_{ph}}{P}$$

where *R* is the responsivity of the detector, and *λ* is the wavelength of the light. Using (1) and (2), the relationship between the *I*_*in*_ and *I*_*ph*_ can be expressed as:

3 $${I}_{ph}\propto R(\lambda , T)P({I}_{in}, T)$$

The CTR of the optocouplers is defined as:

4 $$CTR=\frac{{I}_{out}}{{I}_{in}}$$

where *I*_*out*_ is the output current of the detectors. As the leakage current of the detectors is three orders of magnitude lower than the photocurrent, the output current can be considered as the photocurrent (*I*_*out*_ = *I*_*ph*_). Therefore, the normalized CTR with varying temperatures can be modeled based on (3) and (4).

**Figure 11:**
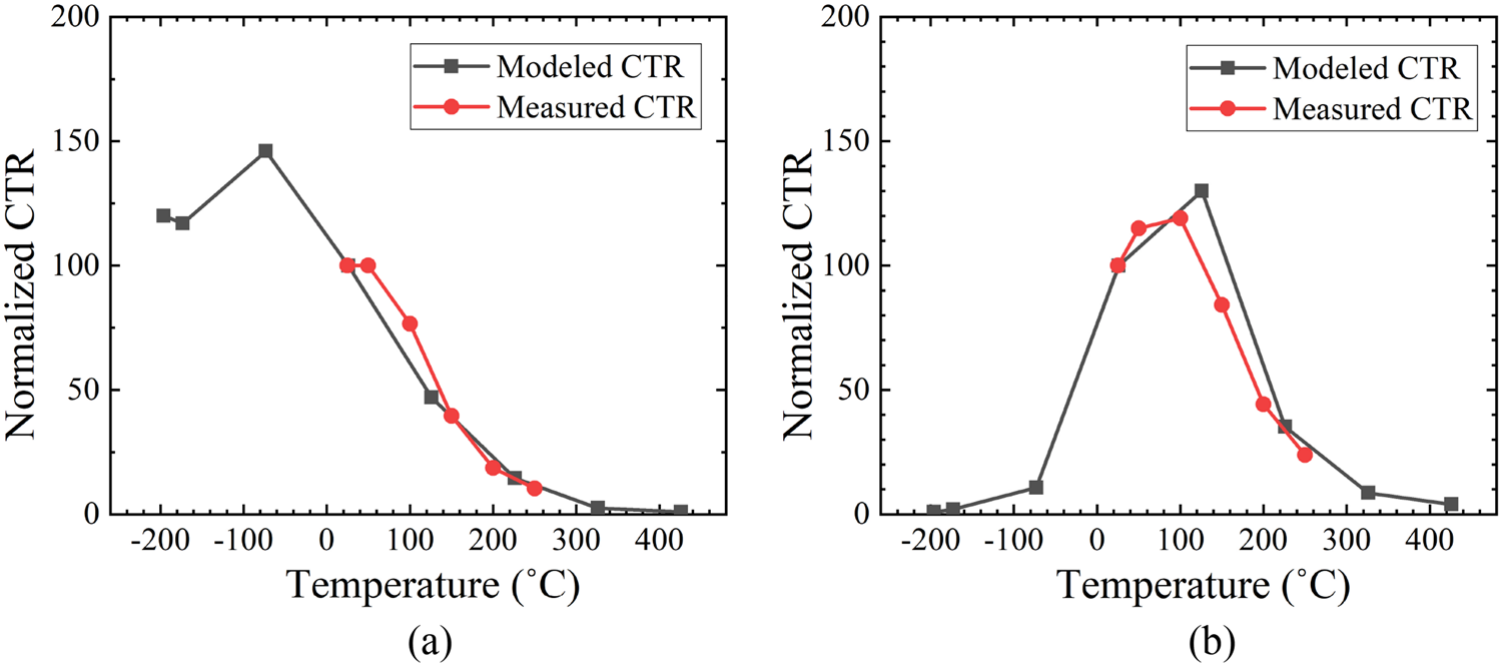
Normalized CTR of (**a**) RD-RD, and (**b**) RD-OPC.

Table 6 shows the normalized integrated EL signal, light wavelength and responsivity of RD-RD and RD-OPC. The results were extracted from previous research, and a detailed experimental setup was discussed^33,34^. The normalized integrated EL signal was extracted at 300 µA injected current. The peak wavelength of the samples (*λ*) increases with the temperature due to the bandgap narrowing effect^34^. The responsivity of the detector was extracted at the corresponding wavelength of the input light. Figure 11a,b show the normalized CTR with varying temperatures of RD-RD and RD-OPC, respectively. The CTR at 25  °C is set as the reference value (i.e., 100). The black line represents the modeled CTR based on (3) and (4), and the red line represents the experimental CTR. As shown in Fig. 11a, the modeled CTR increases with temperature from − 200 to − 75 °C due to the increase of the responsivity. When the temperature is higher than 125 °C, the CTR drops rapidly due to the decrease of the integrated EL signal. The modeled CTR drops to 2.51 and 0.97 when the temperature is 325 °C and 425 °C, respectively. The CTR of RD-OPC increases when the temperature varies from − 200 to 125 °C (Fig. 11b). This is contributed to the enhancement of the responsivity with the increase of temperature. When the temperature is higher than 125 °C, the integrated EL signal decreases rapidly, resulting in the drop of the CTR. The modeled CTR drops to 8.6 and 3.96 when the temperature is 325 °C and 425 °C, respectively.

**Table 6:** Normalized integrated EL signal, light wavelength and responsivity of RD-RD and RD-OPC^33,34^.

*T* (°C)	Normalized *L*_*EL*_ of RD	*λ* (nm) of RD	*R* (A/W) of RD	*R* (A/W) of OPC
− 200	149	606	0.17	2.7 × 10^–4^
− 175	145	607	0.17	5.86 × 10^–4^
− 75	140	617	0.22	0.0033
25	100	630	0.21	0.043
125	44.9	644	0.22	0.14
225	11.1	658	0.22	0.21
325	2.51	672	0.21	0.25
425	1.07	684	0.19	0.28

The photocurrent current and leakage current strongly affect the noise and SNR of the optocouplers. SNR is defined as the ratio of the signal power and noise power, which can be expressed as^30^:

5 $$SNR=\frac{{I}_{ph}^{2}}{B({\sigma }_{th}^{2}+{\sigma }_{sh}^{2}+{\sigma }_{lk}^{2})}$$

where *B* is the bandwidth of the photodetector, and *σ*_th_^*2*^, *σ*_*sh*_^*2*^ and *σ*_*lk*_^*2*^ are thermal noise, shot noise and dark current noise, which can be expressed as:

6 $${\sigma }_{th}^{2}=4kT/{R}_{L}$$

7 $${\sigma }_{sh}^{2}=2q{I}_{ph}$$

8 $${\sigma }_{lk}^{2}=2q{I}_{lk}$$

where *k* is the Boltzmann constant, *T* is temperature, *R*_*L*_ is the load resistance (i.e., 15 kΩ in Fig. 9), *q* is the electron charge, and *I*_*lk*_ is the leakage current. In terms of (6) to (8), the noise components of the optocouplers at 25 °C and 250  °C are summarized in Table 7. As shown in Table 7, for the proposed LTCC-based high-temperature optocouplers, thermal noise and shot noise are the dominant noise at both 25 °C and 250 °C. On the other hand, for the commercial optocoupler (i.e., IL300), dark current noise becomes the dominant noise at 250 °C due to the increase of the leakage current at elevated temperatures.

**Table 7:** Noise components of the optocouplers at 25 °C and 250 °C.

	25 °C			250 °C		
	*σ*_*th*_^*2*^ (W/Hz)	*σ*_*sh*_^*2*^ (W/Hz)	*σ*_*lk*_^*2*^ (W/Hz)	*σ*_*th*_^*2*^ (W/Hz)	*σ*_*sh*_^*2*^ (W/Hz)	*σ*_*lk*_^*2*^ (W/Hz)
RD-RD	1.1 × 10^–24^	1.77 × 10^–24^	6.86 × 10^–30^	1.84 × 10^–24^	2.3 × 10^–25^	1.34 × 10^–28^
RD-OPC	1.1 × 10^–24^	4.29 × 10^–23^	1.46 × 10^–27^	1.84 × 10^–24^	4.36 × 10^–24^	5.12 × 10^–26^
RD-3RD	1.1 × 10^–24^	7.8 × 10^–24^	2.2 × 10^–29^	1.84 × 10^–24^	1.08 × 10^–23^	3.6 × 10^–28^
RD-3OPC	1.1 × 10^–24^	1.08 × 10^–22^	3.42 × 10^–27^	1.84 × 10^–24^	1.92 × 10^–23^	1.97 × 10^–25^
IL300	1.1 × 10^–24^	9.6 × 10^–23^	1.48 × 10^–27^	1.84 × 10^–24^	2.3 × 10^–23^	1.06 × 10^–22^

The response time of RD-3RD is at the same magnitude as RD-RD, while the response time of RD-3OPC is three times higher than RD-OPC. The response time is considered as propagation delay, rise time and fall time. The propagation delay is the time difference between 50% of the final value of input and output. The rise time and fall time are defined as the time difference between the output signal changing from 10 to 90% of its final value. Ideally, the rise time is equal to the fall time, which is defined as^35,36^:

9 $$\tau =\sqrt{{{(2.2\tau }_{RC})}^{2}+{{\tau }_{drift}}^{2}+{{\tau }_{diff}}^{2}}$$

where *τ*_*RC*_ is the RC time constant, *τ*_*drift*_ is the drift time of carriers in the depletion region, and *τ*_*diff*_ is the diffusion time of the carriers. The RC time constant is defined as:

10 $${\tau }_{RC}={R}_{L}{C}_{j}$$

where *R*_*L*_ is the load resistance, and *C*_*j*_ is the junction capacitance of the detectors. The junction capacitance can be expressed as:

11 $${C}_{j}=\frac{{\varepsilon }_{r}{\varepsilon }_{0}A}{{W}_{d}}$$

where *ε*_*r*_ is the dielectric constant of the device, *ε*_*0*_ is the permittivity of free space, *A* is the area of the depletion region, and *W*_*d*_ is the width of the depletion region.

Table 8 shows the measured junction capacitance, RC time constant and rise/fall time of the optocouplers while *R*_*L*_ is 15 kΩ (Fig. 9). The capacitance of 3RD is 52.7 pF, which is around three times higher than the RD (i.e., 18.5 pF). The capacitance of OPC and 3OPC are 387 pF and 1.2 nF, respectively. The junction capacitance of the detectors is proportional to their area. As shown in Table 6, the 2.2*τ*_*RC*_ of RD-RD and RD-3RD is much lower than their rise/fall time. This indicates that the rise time and fall time of RD-RD and RD-3RD are dominated by the drift time and diffusion time of the carriers. Therefore, RD-RD and RD-3RD have the same magnitude of rise/fall time. In addition, for RD-OPC and RD-3OPC, the rise/fall time is equal to 2.2*τ*_*RC*_. This indicates that the rise time and fall time of the RD-OPC and RD-3OPC are dominated by the RC time constant. Thus, the rise/fall time increases three times when an OPC is replaced by three OPCs in parallel, according to (11).

**Table 8:** Junction capacitance, RC time constant and rise/fall time of the optocouplers.

	RD-RD	RD-3RD	RD-OPC	RD-3OPC
*C*_*j*_ (pF)	18.5	52.7	387	1200
2.2*τ*_*RC*_ (µs)	0.61	1.73	12.7	39.6
Rise/fall time (µs)	4	4.6	12.7	40

## Conclusion

High-temperature optocouplers based on LTCC packaging are fabricated and demonstrated as signal galvanic isolation devices for high-temperature applications. The high-temperature optocouplers with various emitters and detectors were fabricated and characterized to identify the coupling efficiency. It is determined that RD-RD and RD-OPC show relatively good optical coupling behaviors. The output current of RD-RD and RD-OPC is 5.5 µA and 134 µA, respectively. Meanwhile, RD-3RD and RD-3OPC are fabricated to improve the output current of the optocouplers. The measurement results show that the RD-3RD and RD-3OPC show output current of 24 µA and 337 µA, respectively. The optocouplers were also characterized over a wide range of temperatures (i.e., 25 °C to 250 °C). The CTR degrades at high temperatures, while the leakage current shows little degradation with varying temperatures. In addition, the response time of the optocouplers is also characterized with varying temperatures. It is observed that the response time shows low temperature sensitivity. RD-RD and RD-3RD have the same magnitude of response time, while RD-3OPC has a response time that is three times higher than RD-OPC. This is because the response time of RD-RD and RD-3RD is dominated by the drift time and diffusion time of the carriers, and the response time of RD-OPC and RD-3OPC is dominated by the RC time constant.

In conclusion, the LTCC-based high-temperature optocouplers (e.g., RD-3OPC and RD-OPC) are promising to operate as galvanic isolation devices up to 250 °C. Utilizing multiple detectors in parallel improves the CTR. However, it also increases the junction capacitance and decreases the response speed. Moreover, the isolation voltage of the optocouplers is related to the distance between the emitter and the detector. Increasing the distance elevates the isolation voltage. However, it may reduce the CTR. Therefore, the trade-off among CTR, response speed and isolation voltage will be investigated in future experiments.
